# Evaluation of a time-resolved singlet oxygen detection system for in vivo photodynamic therapy

**DOI:** 10.1364/BOE.592726

**Published:** 2026-03-27

**Authors:** Vikas Vikas, Baozhu Lu, Brian C. Wilson, Timothy C. Zhu, Robert H. Hadfield

**Affiliations:** 1 James Watt School of Engineering, University of Glasgow, Glasgow G128LT, UK; 2 Department of Radiation Oncology, University of Pennsylvania, Philadelphia, PA 19104, USA; 3 Department of Medical Biophysics, University Health Network/University of Toronto, Toronto M5G 1L7, Canada; 4 vikas.vikas.2@glasgow.ac.uk; 5 Robert.Hadfield@glasgow.ac.uk

## Abstract

Singlet oxygen (^1^O_2_) is the primary cytotoxic agent in Type-II photodynamic therapy (PDT). Single-photon avalanche diode (SPAD)-based time-resolved singlet oxygen luminescence detection (TSOLD) systems enable direct detection of the near-infrared ^1^O_2_ phosphorescence (∼1270 nm) during PDT, offering a powerful tool for treatment monitoring. However, the efficiency of detection depends strongly on the acquisition parameters. Here, we present a comprehensive evaluation of a TSOLD system with 630 nm and 690 nm nanosecond pulsed diode lasers developed for *in vivo* PDT monitoring. The influence of acquisition time (1–300 s), SPAD dead time (5-40 µs), and temporal bin width (1–100 ns) on the fidelity of ^1^O_2_ lifetime measurements and signal-to-noise ratio (SNR) was quantitatively investigated. Measurements were performed using liquid phantoms containing the photosensitizers Photofrin or benzoporphyrin derivative, with acquisition time further validated *in vivo* in murine tumor models. The results demonstrate that both acquisition time and detector dead time significantly affect the signal *vs.* time histogram shape and the accuracy of the ^1^O_2_ and photosensitizer triplet-state lifetime measurements, while an optimal bin width minimizes photon-count noise without compromising temporal resolution. The optimized parameters enable reliable ^1^O_2_ lifetime extraction within 100 s from the mouse model *in vivo*. This systematic evaluation establishes quantitative design guidelines for compact TSOLD systems tailored to *in vivo* applications.

## 1. Introduction

Photodynamic therapy (PDT) is a clinically approved, minimally invasive treatment modality for both cancers and non-malignant diseases. It involves three essential components: the photosensitizer concentration, light of an appropriate wavelength to activate the photosensitizer, and molecular oxygen in the tumor region [1,2]. Upon light activation, the photosensitizer transfers energy to surrounding oxygen molecules, producing reactive oxygen species (ROS), predominantly singlet oxygen (^1^O_2_), the key cytotoxic agent responsible for inducing tumor cell apoptosis, necrosis and vascular shutdown [1,3–5]. PDT provides numerous benefits compared to traditional therapies, such as spatial selectivity, repeatability, and minimal systemic toxicity. It has been effectively used to treat skin, head and neck, lung, and gastrointestinal cancers, as well as ocular and antimicrobial conditions [6,7].

Despite its clinical effectiveness, the outcomes of photodynamic therapy (PDT) can vary significantly among patients. This variability is attributed to the intricate interplay among factors such as the concentration of the photosensitizer, the fluence of light, and the local levels of oxygen [8,9]. Conventional dosimetry in PDT often relies on simply measuring either the administered light dose and/or the administered photosensitizer dose. However, this approach does not accurately reflect the actual cytotoxic species generated during treatment. Hence, reactive oxygen species dosimetry, particularly the direct monitoring of ^1^O_2_, is regarded as the gold standard for predicting treatment efficacy [10–12]. However, quantifying ^1^O_2_ *in vivo* is challenging due to its weak phosphorescence emission at 1270 nm (with a quantum yield of approximately 10^−7^) and its short-lived nature, with a lifetime of microseconds or less [13,14]. The ability to ^1^O_2_ monitor in real time would provide an objective and specific molecular indicator of therapeutic response. Several techniques, such as multispectral singlet oxygen luminescence dosimetry (MSOLD), luminescence micro spectroscopy, and continuous SOLD (CSOLD) have been explored for singlet oxygen detection, as well as chemical probes, near-infrared sensors, and indirect fluorescence or oxygen-consumption assays [15–20]. However, its broader clinical adoption has been limited by challenges related to low signal levels, detector non-idealities, and parameter-dependent fitting instability. Among these approaches, *time-resolved singlet oxygen luminescence detection (TSOLD)* uniquely enables direct detection of the 1270 nm phosphorescence signal with temporal discrimination of singlet oxygen and photosensitizer triplet dynamics [21–23]. TSOLD measures the delayed 1270 nm luminescence following pulsed excitation and allows quantitative extraction of ^1^O_2_ lifetime and yield, both of which correlate with tissue oxygenation and photochemical efficiency [11,24]. However, the extremely low photon emission rate requires sensitive detection systems with precise temporal resolution. In the initial development, TSOLD has relied on photomultiplier tubes (PMTs) or cooled InGaAs detectors coupled to time-correlated single-photon counting (TCSPC) modules [25,26]. While these systems have provided valuable insights into PDT mechanisms, their size, cost and complexity have limited their clinical translation. Recent advances in single-photon avalanche diode (SPAD) technology have transformed photon-counting detection [27]. SPADs offer high quantum efficiency in the near-infrared, compact form factors and low power consumption, enabling portable TSOLD systems suitable for preclinical or bedside use [28,29]. Moreover, modern TCSPC electronics integrated with field-programmable gate arrays (FPGAs) provide sub-nanosecond timing resolution and flexible acquisition control [30]. These developments make it feasible to design lightweight, portable, and cost-effective TSOLD instruments for *in vivo* monitoring.

Achieving accurate quantification of ^1^O_2_ lifetime requires careful optimization of the measurement parameters that influence signal quality. The shape and accuracy of the time-resolved luminescence histogram depend on several factors, including the detector dead time, the temporal bin width and the total acquisition time [31]. Improper selection of these parameters can lead to distorted decay profiles, poor signal-to-noise ratio (SNR), or erroneous lifetime estimation. While individual aspects of detector performance have been discussed in prior studies, a systematic evaluation of these acquisition parameters for portable SPAD-based TSOLD systems, particularly under *in vivo* conditions, remains lacking.

To address this gap, we present a comprehensive analysis of a compact time-resolved singlet oxygen luminescence detection (TSOLD) system developed in-house for *in vivo* photodynamic therapy (PDT) studies. The system utilizes nanosecond pulsed diode lasers operating at 630 nm and 690 nm that align with the absorption peaks of two clinically relevant photosensitizers, Photofrin and benzoporphyrin derivative (BPD). The detection of the weak 1270 nm emission is carried out using an InGaAs SPAD paired with customized optical filters and an ultrafast TCSPC module.

We systematically investigated the effects of various acquisition parameters, including acquisition time ranging from 1 to 300s, SPAD dead time from 5 to 40 µs, and temporal bin width from 1 to 100 ns, on the accuracy of ^1^O_2_ lifetime and signal-to-noise ratio (SNR) measurements. Experiments were conducted using liquid tissue-simulating phantoms containing BPD or Photofrin, followed by *in vivo* validation in murine tumor models. This work provides the first detailed quantitative assessment of these parameters in a portable TSOLD platform. Our findings highlight the relative importance of each acquisition factor and establish optimized operating conditions for reliable *in vivo* detection. The resulting design guidelines will be useful for researchers developing compact singlet oxygen dosimetry systems and advancing PDT towards precise, feedback-controlled therapeutic applications.

## 2. Methods

### 2.1. TSOLD system

**Fig. 1.**
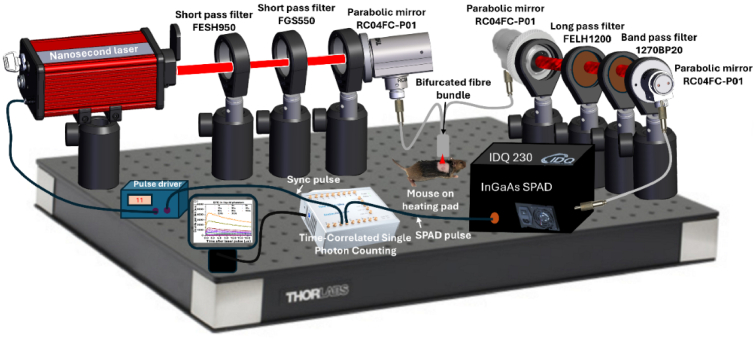
Schematic of TSOLD system (not to scale). A nanosecond pulsed diode laser is coupled into a bifurcated fiber via a parabolic mirror and passes through short-pass filters to illuminate the sample (mouse tumor *in vivo* or liquid sample in a cuvette). The emitted ^1^O_2_ phosphorescence at 1270 nm is collected through the same fiber arm and directed to an InGaAs SPAD detector via a 1270 nm band-pass filter. The TCSPC module records synchronized photon arrival times using the laser sync signal and SPAD output to generate time-resolved ^1^O_2_ histograms.

A schematic of the in-house developed TSOLD system is shown in Fig. 1. The system embeds a pulsed diode laser with a pulse driver, a combination of short-, long- and band-pass filters, a parabolic mirror with fiber coupler, a bifurcated fiber bundle, InGaAs SPAD, and an ultrafast time correlated single photon counter.

In this setup, a pulsed diode laser with wavelength 630 nm (NPL64C: Thorlabs, Newton, NJ, USA) and 685 nm (LP685-SF15: Thorlabs, Newton, NJ, USA), pulse width 129 ns, and repetition rate 50 kHz, is selected to match the treatment wavelength used for Photofrin and BPD, respectively, aiming for maximum penetration of light in tissue. The output was coupled into one end of a bifurcated fiber (200 µm core, 0.39 numerical aperture, FCR-7UVIR200-2-ME-FC, Avantes, USA) and delivered to the sample through short-pass (FESH0950, Thorlabs, Newton, NJ, USA) and band-pass (FWHM 304–785 nm) (FGS550, Thorlabs, Newton, NJ, USA) filters. The output power of the laser was set to give a maximum clinical fluence rate on the sample of 150 mWcm^-2^. The luminescence emitted at ∼1270 nm was collected using fiber-based epi-illumination, spectrally filtered by a long-pass filter (λ > 1200 nm, FELH1200, Thorlabs, USA) and a narrowband filter with transmission between 1260 and 1280 nm, OD ≥ 6 (1270BP20, Omega Optical, Brattleboro, VT, USA). The light was then coupled into a multimode fiber connected to an InGaAs SPAD detector (ID Quantique ID230, IDQ, Geneva, Switzerland) operating in free-running mode. The output pulses were sent into a TCSPC module (Time Tagger Ultra, Swabian Instruments, Germany) with programmable bin width (1–100 ns) and acquisition time control, generating single photon counting-based time histograms.

### 2.2. Phantom and mouse preparation for singlet oxygen detection

The liquid phantom formulation used in this study was based on the previously published and experimentally validated protocol reported by Yang et al., which has been used for singlet oxygen detection studies [20]. Liquid phantoms with tissue-like optical properties were prepared using 0.6% Intralipid (20% w/v, Fresenius-Kabi, USA) ( $\mu_{s}^{\prime }$ = 8 cm^−1^) and 0.002% India ink (*μ*_a_ = 0.1 cm^-1^) in ethanol. Photofrin was added at a concentration of 5 mg/kg or BPD was added at a concentration of 2 mg/kg in a quartz cuvette (path length 10 mm) with a screw cap (CV10Q35EP, Thorlabs, Newton, NJ, USA). To optimize the ^1^O_2_ luminescence measurements, the optical fiber tip was placed close to the phantom surface. All measurements were conducted immediately after preparation and under consistent laboratory conditions to minimize variability and ensure stability during acquisition.

**Fig. 2.**
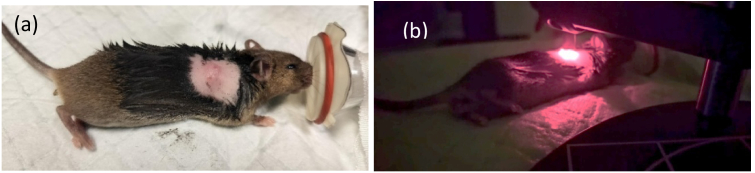
Photograph of tumor-bearing C3H mice (a) with visible subcutaneous RIF tumor (∼5 mm diameter) prior to treatment, and (b) under 630 nm laser illumination at the tumor site during TSOLD measurements.

For the *in-vivo* study, female C3H mice (5-8 weeks old) bearing subcutaneous RIF tumors (∼2–5 mm diameter) were injected intravenously with Photofrin (5 mg/kg body weight) or BPD (2 mg/kg). TSOLD measurements were performed under anaesthesia at time intervals of 3 h (Photofrin) or 24 h (BPD) post injection. The measurements were performed using the TSOLD fiber-optic system with the fiber tip placed close to the surface of the skin overlying the tumor. The background signals from *un-injected* control mice were subtracted from ^1^O_2_ signal luminescence. All procedures were approved by the Institutional Animal Care and Use Committee (IACUC) of the University of Pennsylvania, with animal care supervised by the U. Penn. Laboratory Animal Resources. During treatment, the mice were anesthetized by isoflurane and maintained at 37 °C on a temperature-controlled heating pad (Fig. 2).

### 2.3. Optimization of experimental parameters for efficient singlet oxygen detection

In order to ensure accurate and high-fidelity detection of ^1^O_2_ phosphorescence, key acquisition parameters of the TSOLD system were systematically optimized. Three critical parameters were varied to evaluate their impact on signal quality, photon statistics and extracted lifetime accuracy.

(a). **Acquisition time:** Extended acquisition durations ranging from 1 to 300 s were used to investigate how total photon counts affect the signal-to-noise ratio (SNR) and the statistical reliability of lifetime fitting for Photofrin and BPD in liquid phantoms and tumor. Shorter acquisition times allowed for rapid measurements that are suitable for *in vivo* use, while longer durations enhanced the signal-to-noise and reduced uncertainty in the calculation of singlet oxygen and triplet-state lifetimes.
(b). **Time bin width:** The FPGA-based timing electronics were configured to investigate the trade-off between temporal resolution and noise sensitivity. Narrow time bins improved precision in lifetime measurements but necessitated longer acquisition times to obtain enough counts per bin. In this study, the time bin width was varied from 1 to 100 ns to determine the optimal width, using a 100 s acquisition time for 5 mg/kg Photofrin in liquid phantoms.
(c). **SPAD dead time:** The internal quenching control of the short InGaAs SPAD was adjusted to evaluate its impact on photon pile-up, histogram distortion and effective count rate. The dead time of the SPAD was modified from 5 to 40 µs to ensure linear photon detection under different signal intensities for 5 mg/kg Photofrin in liquid phantoms with an acquisition time of 100 s.

### 2.4. Data processing and analysis singlet oxygen lifetime

The parameters of the TSOLD system, including acquisition time, dead time and bin width for time acquisition, play a significant role in estimating the lifetime of ^1^O_2_ and its effects. The lifetime of ^1^O_2_ is determined using bi-exponential curve fitting of the measured time histogram, as described in Eq. (1) [16,22]. This fitting was performed after subtracting the background signal.

(1) $$[1_{O_{2}}](t)=A.\;\frac{\tau_{D}}{\tau_{T}-\tau_{D}}\;\left(\exp\left(\frac{-t}{\tau_{T}}\right)\;-\exp\left(\frac{-t}{\tau_{D}}\right)\;\right)$$

Where, A = $N\sigma [S_{0}]\varphi_{D\;},$ [^1^O_2_](t) is the concentration of singlet oxygen as a function of time produced on illumination of *N* photons per cm^2^, *σ* is the ground-state photosensitizer absorption cross-section at the excitation wavelength, $\varphi_{D}$ is the photosensitizer singlet-oxygen quantum yield, [*S*_0_] is the photosensitizer concentration, and *τ_D_* and *τ_T_* are the lifetimes of singlet oxygen and triplet-state photosensitizer, respectively [16]. This approach enabled accurate extraction of ^1^O_2_ lifetime, despite the presence of overlapping fast-decay signals from tissue scattering. For estimation of lifetimes *in-vivo*, the bi-exponential curve fitting was done assuming that *τ_D_* << *τ_T_* [11,29].

## 3. Results

**Fig. 3.**
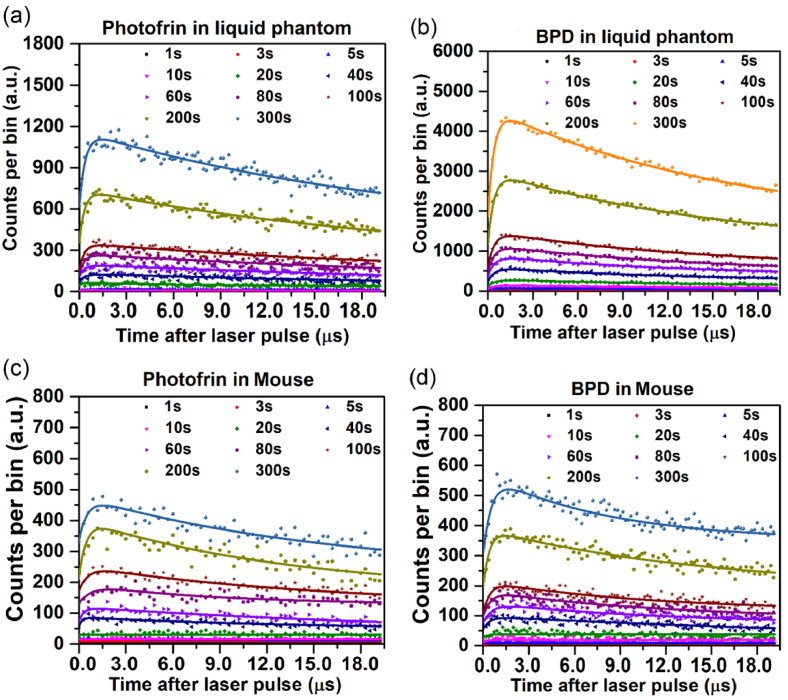
Acquisition time dependence of ^1^O_2_ photon counting time curves (dots: measured, lines: exponential fits) for (a) 5 mg/kg Photofrin in liquid phantom, (b) 2 mg/kg BPD in liquid phantom, (c) 5 mg/kg Photofrin in tumor, and (d) 2 mg/kg BPD in tumor recorded at 1270 nm.

Acquisition time dependent ^1^O_2_ photon counting decay curves for Photofrin and BPD in liquid phantoms and in mice recorded at 1270 nm for integration times ranging from 1 to 300 s are shown in Fig. 3. Table 1 and 2 summarize the fitted lifetimes of ^1^O_2_ and triplet-state photosensitizer, along with the corresponding coefficients of determination (R^2^) and signal-to-noise ratio (SNR) values.

**Table 1.** Lifetimes of ^1^O_2_ (τ_D_), and triplet-states photosensitizer (τ_T_) in liquid phantoms with corresponding R^2^ and SNR values from biexponential fitting of TSOLD data.

Acquisition time	Photofrin in liquid phantom				BPD in liquid phantom			

	*τ_D_* (µs)	*τ_T_* (µs)	R^2^ Values	SNR ratio	*τ_D_* (µs)	*τ_T_* (µs)	R^2^ Values	SNR ratio
**1 s**	0.24	0.10	0.0378	1.36	0.35	34.1	0.1951	4.1
**3 s**	19.3	0.31	0.0921	3.13	0.42	36.6	0.2781	6.94
**5 s**	16.6	0.46	0.1127	6.25	0.53	24.8	0.2939	8.35
**10 s**	6.94	0.30	0.0816	6.02	11.88	0.40	0.7334	20.09
**20 s**	5.90	0.20	0.2974	13.58	11.81	0.38	0.8666	23.63
**40 s**	12.92	0.25	0.4865	17.45	13.25	0.25	0.9203	26.72
**60 s**	13.63	0.23	0.5601	18.79	13.53	0.23	0.9456	28.41
**80 s**	13.51	0.22	0.6363	20.51	13.23	0.22	0.9611	29.82
**100 s**	13.06	0.25	0.672	21.47	13.56	0.17	0.9668	30.52
**200 s**	13.20	0.24	0.8168	23.6	13.72	0.18	0.978	32.25
**300 s**	13.15	0.22	0.867	25.81	13.31	0.21	0.9875	34.72

**Table 2.** Lifetimes of ^1^O_2_ (***τ_D_***), and triplet states photosensitizers (τ_T_) in mouse (*in vivo)* with corresponding R^2^ and SNR values from biexponential fitting of TSOLD data.

Acquisition time	Photofrin in mouse				BPD in mouse			

	*τ_D_* (µs)	*τ_T_* (µs)	R^2^ Values	SNR ratio	*τ_D_* (µs)	*τ_T_* (µs)	R^2^ Values	SNR ratio
**1 s**	1.10	1.20	0.0025	1.84	0.52	40.90	0.0362	1.55
**3 s**	1.75	1.75	0.1151	2.8	0.56	20.79	0.1373	2.55
**5 s**	2.90	5.10	0.0791	3.96	2.60	2.59	0.2182	3.26
**10 s**	2.38	1.55	0.1292	3.89	1.31	5.98	0.2837	4.61
**20 s**	2.90	4.10	0.2639	6.07	2.36	3.07	0.4267	6.31
**40 s**	1.70	6.40	0.3107	9.29	0.88	3.26	0.5773	8.72
**60 s**	1.60	7.81	0.4502	10.92	0.33	8.06	0.6026	10.65
**80 s**	0.43	8.10	0.5723	10.75	0.27	7.71	0.6208	11.52
**100 s**	0.30	9.13	0.5669	11.94	0.23	7.31	0.6926	14.09
**200 s**	0.25	8.72	0.8165	15.07	0.22	8.88	0.8127	16.79
**300 s**	0.26	9.78	0.8224	18.41	0.28	8.69	0.8401	21.19

Photon counts and signal-to-noise ratio (SNR) increased approximately as the square root of the acquisition time in TSOLD measurements. Based on the analysis of R^2^ and SNR values, the detection sensitivity and lifetime precision of ^1^O_2_ improved significantly for acquisition times exceeding 40 s in liquid phantoms, whereas *in vivo* measurements longer than 100 s to achieve comparable reliability. Consistently high R^2^ values (∼0.80) and favourable SNRs (>15) across all datasets confirm the robustness and reproducibility of the TSOLD approach for accurate *in vivo* ^1^O_2_ detection with acquisition time >= 200 s [32].

**Fig. 4.**
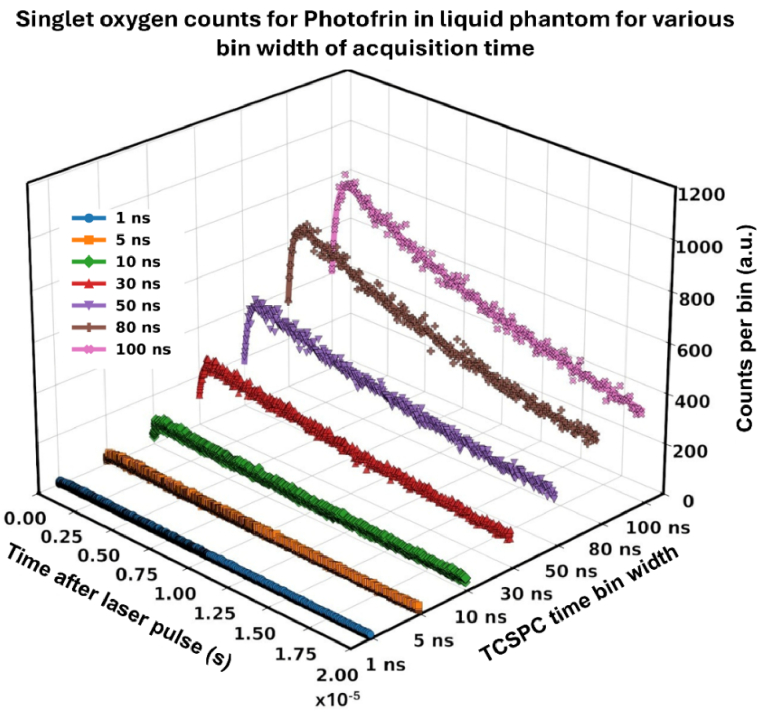
^1^O_2_ photon-counting curves (dots: measured data; lines: exponential fits) for 5 mg/kg Photofrin in liquid phantom, recorded at 1270 nm using TCSPC with time bin widths ranging from 1 to 100 ns with 100 s integration time.

Figure 4 presents the time-resolved histogram of ^1^O_2_ counts for 5 mg/kg Photofrin in liquid phantoms for various TCSPC time bin widths using the 630 nm laser at an irradiance of 150 mW/cm^2^. The calculated triplet-state of Photofrin and ^1^O_2_ in liquid phantoms, derived from bi-exponential fitting of TSOLD data, along with their corresponding R^2^ and SNR values, are presented in Table 3.

**Table 3.** ^1^O_2_ and Photofrin triplet-state lifetimes obtained from bi-exponential fitting of the measured TSOLD time curves for TCSPC time bin widths of 1 to 100 ns, along with corresponding R^2^ and SNR values.

TCSPC bin width	*τ_D_* (µs)	*τ_T_* (µs)	R^2^ Values	SNR ratio
**1 ns**	15.18	0.19	0.3433	2.90
**5 ns**	12.97	0.25	0.6196	5.28
**10 ns**	14.89	0.16	0.7197	7.74
**30 ns**	14.11	0.20	0.8245	13.88
**50 ns**	13.2	0.26	0.9565	21.51
**80 ns**	13.65	0.32	0.9624	21.88
**100 ns**	14.16	0.43	0.9821	24.56

**Fig. 5.**
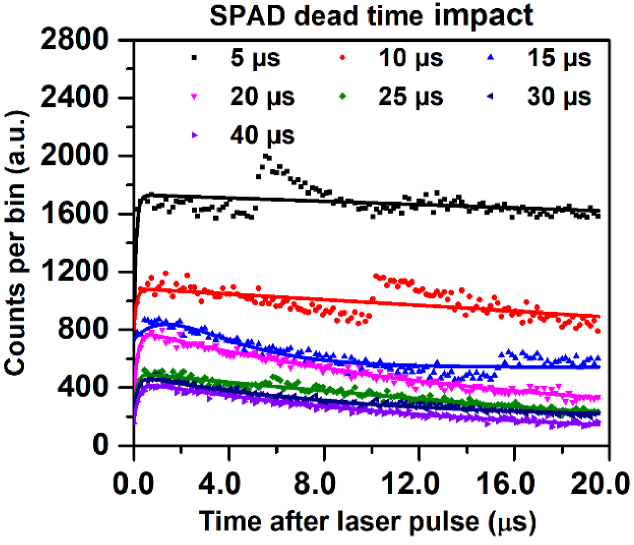
TSOLD photon-counting decay curves (dots: measured data; lines: fitted curves) recorded at 1270 nm for SPAD dead times of 5-40 µs, acquired with 100 s integration.

The bin width–dependence study revealed that increasing the bin width led to higher photon counts per bin, accompanied by improved R^2^ and SNR. However, at smaller bin widths (<30 ns), although the temporal resolution was enhanced, the SNR degraded, making accurate extraction of the singlet oxygen lifetime difficult. Conversely, excessively coarse binning (>50 ns) resulted in under-sampling of the decay curve and systematic overestimation of the lifetimes. Based on these observations, an optimal bin width of 50 ns was selected for subsequent experiments. The SPAD dead time dependent time measurement for Photofrin in liquid phantoms is shown in Fig. 5.

From Fig. 5, it is evident that the histogram shape strongly depends on the SPAD dead time, which plays a critical role in the accurate detection of singlet oxygen signals. The corresponding lifetimes are summarized in Table 4.

**Table 4.** Fitted ^1^O_2_ and Photofrin triplet-state lifetimes obtained from bi-exponential fitting of TSOLD decay curves for SPAD dead times of 5-40 µs, along with corresponding R^2^ and SNR values.

SPAD dead time	*τ_D_* (µs)	*τ_T_* (µs)	R^2^ Values	SNR ratio
**5 µs**	2016	2015.0	0.1958	2.90
**10 µs**	8214	0.08	0.2145	6.28
**15 µs**	2.18	2.14	0.6578	13.12
**20 µs**	13.21	0.22	0.9345	22.88
**25 µs**	56.00	0.12	0.845	16.50
**30 µs**	9.16	0.16	0.8645	16.45
**40 µs**	13.45	0.23	0.9214	21.45

The SPAD dead time directly impacts the generated histogram as well as the calculation of the ^1^O_2_ and triplet-state lifetimes. When the SPAD dead time was set equal to approximately one (20 µs) or two (40 µs) times the laser pulse repetition interval, the acquired signals exhibited clear biexponential decays characteristic of singlet oxygen emission allowing reliable lifetime extraction. The unusually large lifetime values obtained at a SPAD dead time of 5 µs do not reflect the intrinsic singlet oxygen or triplet-state dynamics. Instead, they arise from histogram distortion caused by detector recovery limitations and photon pile-up effects. At this short dead time, which is substantially smaller than the 20 µs laser pulse period (50 kHz repetition rate), incomplete detector recovery between excitation cycles leads to non-linear photon counting and deformation of the early decay profile. Consequently, the biexponential fitting routine attempts to model a distorted histogram, resulting in artificially inflated lifetime constants. These results highlight the importance of matching detector dead time to the excitation repetition rate to ensure physically meaningful lifetime extraction. In contrast, shorter or longer dead times relative to the pulse repetition interval led to histogram distortion and temporal broadening, which degraded the fidelity of lifetime determination.

## 4. Discussion

The present study provides a systematic evaluation of the instrument and acquisition parameters that govern the accuracy and fidelity of ^1^O_2_ detection using a portable SPAD-based TSOLD system. Although TSOLD is widely recognized as the most direct and specific method for PDT dosimetry, its practical implementation has been hindered by the weak near-infrared phosphorescence signal at 1270 nm and the need for precise time-correlated single-photon counting conditions. By systematically optimizing acquisition time, TCSPC bin width and SPAD dead time, this work establishes quantitative guidelines for achieving reliable lifetime estimation under both phantom and *in vivo* conditions.

In this study, excitation wavelengths of 630 nm and 690 nm were intentionally selected because they correspond to the primary clinical activation wavelengths with higher penetration depth for the FDA-approved photosensitizers Photofrin and benzoporphyrin derivative (BPD), respectively. These wavelengths are widely used in clinical photodynamic therapy protocols and ongoing translational studies. Although not surprising, a key observation is that the acquisition time critically affects the signal-to-noise ratio (SNR) and the precision of lifetime retrieval. Photon counts and SNR increased with the square root of the integration time, improving the stability of the exponential fits. For liquid phantoms, acquisition times exceeding 40 s yielded sufficiently high SNR (>15) and robust R^2^ values (>0.8), ensuring accurate lifetime determinations. However, in tumor *in-vivo* longer integration times (>100 s) were required to achieve comparable measurement reliability. These findings highlight that acquisition time must be adapted to the measurement conditions in order to maintain adequate photon statistics for meaningful bi-exponential fitting. The optimized 100 s integration period represents a practical compromise between measurement fidelity and experimental feasibility during PDT monitoring

The TCSPC bin width was also found to be a determining factor in balancing temporal resolution against statistical noise. Narrow bin width (<30 ns) enhanced temporal precision but resulted in low photon counts per bin, leading to poor SNR and unstable fits. Conversely, coarse binning (>50 ns) introduced under-sampling and artificial broadening of the decay profile, thereby systematically overestimating ^1^O_2_ lifetimes. An optimal bin width of 50 ns was identified, yielding smooth histograms, good R^2^ values, and reproducible lifetime estimates without compromising timing resolution. These results emphasize that TCSPC configuration must be carefully tuned for each detection geometry to preserve the temporal fidelity of the weak ^1^O_2_ signal.

Furthermore, the SPAD dead time significantly influenced the temporal profile of the histogram and lifetime accuracy. When the dead time was comparable to one or two times the selected laser pulse period (20 or 40 µs for a 50 kHz repetition rate), the histograms exhibited well-defined biexponential decays suitable for lifetime fitting. However, shorter or excessively long dead times produced histogram distortion and intensity pile-up effects, leading to misestimation of decay constants. This relationship underscores the importance of synchronizing SPAD recovery dynamics with excitation pulse timing to prevent photon loss and temporal bias in TSOLD measurements. The 50 kHz repetition rate (20 µs pulse period) was selected because it provides higher average excitation power from the nanosecond diode laser while still fully accommodating the singlet oxygen decay within a single excitation cycle. At lower repetition rates, the reduced average excitation power would decrease photon yield, potentially requiring longer acquisition times to achieve an adequate signal-to-noise ratio.

An additional advantage of the present TSOLD platform is its inherent flexibility to accommodate emerging photosensitizers with diverse absorption characteristics. The use of interchangeable nanosecond diode laser sources allows straightforward adjustment of the excitation wavelength to match the absorption maxima of next-generation photosensitizers, including red-shifted and activatable agents developed for deep-tissue PDT [16,23,29]. Optimizing the dead time of the SPAD and adjusting the acquisition parameters significantly enhances histogram integrity, reduces photon pile-up, and improves the accuracy of biexponential decay fitting. These improvements directly strengthen temporal resolution by preserving the true kinetics of both singlet oxygen and triplet states. Additionally, enhanced signal-to-noise performance increases detection sensitivity, especially in low-photon conditions. Together, these refinements support more reliable real-time monitoring of 1O2 dynamics during photodynamic therapy and facilitate the practical use of compact TSOLD systems in clinical settings.

The present *in vivo* experiments were intended as a feasibility demonstration rather than a comprehensive biological validation study. Representative luminescence spectra, full kinetic analyses, quantitative ^1^O_2_ yield estimation, and correlation with therapeutic outcome were beyond the scope of this work. Future studies will focus on calibrated quantification of singlet oxygen production, acquisition of complete decay kinetics *in vivo*, and systematic correlation with treatment response. Additionally, translation to deeper tissues will require further optimization to address optical attenuation, background luminescence, and signal sensitivity under clinically relevant conditions.

## 5. Conclusions

Collectively, these findings demonstrate that careful tuning of acquisition parameters such as acquisition time, bin width and dead time enables robust extraction of ^1^O_2_ lifetimes from compact, field-deployable TSOLD instruments. The optimized conditions reported here (100 s acquisition, 50 ns bin width, 20 µs dead time) provide a strong foundation for portable and clinically adaptable systems for PDT dosimetry. By bridging the gap between laboratory-grade “gold standard” setups and miniaturized devices for future clinical use, this work advances the practical implementation of direct ^1^O_2_ monitoring for personalized photodynamic therapy as compared to previously-reported PMT-based TSOLD setups [33–35].

## Data Availability

Data underlying the results presented in this paper are available in the University of Glasgow Enlighten Research Data Repository [36].
